# Intra-articular vs. intravenous administration: a meta-analysis of tranexamic acid in primary total knee arthroplasty

**DOI:** 10.1186/s13018-020-02119-1

**Published:** 2020-12-02

**Authors:** Jin Li, Ruikang Liu, Saroj Rai, Renhao Ze, Xin Tang, Pan Hong

**Affiliations:** 1 Department of Orthopaedic Surgery, Union Hospital, Tongji Medical College, Huazhong University of Science and Technology, Wuhan, 430022 China; 2 First School of Clinical Medicine, Tongji Medical College, Huazhong University of Science and Technology, Wuhan, China; 3 Department of Orthopaedics and Trauma Surgery, National Trauma Center, National Academy of Medical Sciences, Mahankal, Kathmandu, Nepal

**Keywords:** Tranexamic acid, Total knee arthroplasty, Intra-articular administration, Intravenous administration

## Abstract

**Background:**

The optimal dosage and administration approach of tranexamic acid (TXA) in primary total knee arthroplasty (TKA) remains controversial. In light of recently published 14 randomized controlled trials (RCTs), the study aims to incorporate the newly found evidence and compare the efficacy and safety of intra-articular (IA) vs. intravenous (IV) application of TXA in primary TKA.

**Methods:**

PubMed, Embase, Web of Science, and Cochrane Library were searched for RCTs comparing IA with IV TXA for primary TKA. Primary outcomes included total blood loss (TBL) and drain output. Secondary outcomes included hidden blood loss (HBL), hemoglobin (Hb) fall, blood transfusion rate, perioperative complications, length of hospital stay, and tourniquet time.

**Result:**

In all, 34 RCTs involving 3867 patients were included in our meta-analysis. Significant advantages of IA were shown on TBL (MD = 33.38, 95% CI = 19.24 to 47.51, *P* < 0.001), drain output (MD = 28.44, 95% CI = 2.61 to 54.27, *P* = 0.03), and postoperative day (POD) 3+ Hb fall (MD = 0.24, 95% CI = 0.09 to 0.39, *P* = 0.001) compared with IV. There existed no significant difference on HBL, POD1 and POD2 Hb fall, blood transfusion rate, perioperative complications, length of hospital stay, and tourniquet time between IA and IV.

**Conclusion:**

Intra-articular administration of TXA is superior to intravenous in primary TKA patients regarding the performance on TBL, drain output, and POD3+ Hb fall, without increased risk of perioperative complications. Therefore, intra-articular administration is the recommended approach in clinical practice for primary TKA.

## Background

Total knee arthroplasty (TKA) is a common major orthopedic surgery, and the demand is still increasing due to human longevity and large population suffering from knee osteoarthritis (OA) around the world [1, 2].

TKA is an effective choice for end-stage OA [3]. But it is a major operation especially for the geriatric population, and the postoperative reduced hemoglobin (Hb) might require blood transfusion and potentially result in delayed physical rehabilitation, longer hospital stay, and higher medical cost [4].

Tranexamic acid (TXA) has been widely used in many orthopedic surgeries for controlling blood loss [5]. Its safety and efficacy has been validated by many studies [6–8]. However, the optimal administration approach for primary TKA remains to be investigated. Oral administration and intravenous (IV) administration have been validated as an effective approach, but there are potential risks of thromboembolic complications [9, 10]. Besides, intra-articular (IA) administration provides a maximum concentration at the bleeding site with limited systemic influence [11].

Gianakos et al. [12] published the latest meta-analysis on IA vs. IV in 2018, and it demonstrated the superiority of IA over IV administration. However, with the publication of 14 new randomized controlled trial (RCT) results thereafter [13–26], it is imperative to perform a new meta-analysis to corroborate or repudiate the conclusion of Gianakos et al., which is the purpose of our study.

## Methods

Our meta-analysis was conducted in accordance with the guidelines of the PRISMA (Preferred Reporting Items for Systematic Reviews and Meta-Analyses statement) [27]. We did not publish a protocol for this study.

### Literature search

Four electronic databases including PubMed, Embase, Web of Science, and Cochrane Library were searched. Searching was conducted until April 20, 2020, with the following search terms: (“tranexamic acid” OR “TXA”) AND (“total knee arthroplasty” OR “total knee replacement” OR “TKA” ). Literatures were limited to English publication. All studies were full text available. Unpublished investigations were not included.

### Selection criteria

Two independent reviewers performed the search, removed duplicate records, reviewed the titles and abstracts, and identified studies as included, excluded, or uncertain. Full-text articles were reviewed to determine eligibility if identified uncertain. Disagreements were discussed with a third reviewer.

We retrieved all RCTs that compared IA with IV administration of TXA in patients receiving primary TKA. Inclusion criteria were (1) patients who underwent primary TKA, (2) comparative studies of IA vs. IV administration of TXA, (3) availability of full text, and (4) English publications. Exclusion criteria were (1) non-cohort studies, (2) retrospective cohort studies, (3) reviews, and (4) unpublished studies.

### Data extraction

The following data were extracted: characteristics of study (design, country, no. of patients, age, sex, body mass index, follow-up, and conclusion), method of administration and operation (IV or IA dosage, type of operation, and surgical approach), and surgical protocols (thromboprophylaxis, DVT screening, prosthetic properties, blood transfusion protocol, tourniquet application, and drainage).

Primary outcomes included total blood loss (TBL), which was calculated by the Gross formula or Hb balance method [28, 29], and drain output. Secondary outcomes included hidden blood loss (HBL), Hb fall, blood transfusion rate, and perioperative complications including deep vein thrombosis (DVT), pulmonary embolism (PE), wound infection, and other vascular events. The duration of tourniquet application and length of hospital stay were also recorded and analyzed. Missing data were obtained from corresponding authors if possible.

### Quality assessment

We assessed the qualities of included studies according to the criteria of the *Cochrane Handbook for Systematic Reviews of Interventions* [30]. The strength of evidence for each major outcome was evaluated according to the 8-point modified Jadad scale (Table 1) [31]. A study scoring above 4 was considered qualified. A study scoring above or equal to 7 was considered as high-quality evidence.

**Table 1:** Modified Jadad scale

Item assessed	Score
Was the study described as randomized?	
Yes	+ 1
No	0
Was the method of randomization appropriate?	
Yes	+ 1
No	− 1
Not described	0
Was the study described as blinded?	
Yes	+ 1
No	0
Was the method of blinding appropriate?	
Yes	+ 1
No	− 1
Not described	0
Was there a description of withdrawals and dropouts?	
Yes	+ 1
No	0
Was there a clear description of the inclusion/exclusion criteria?	
Yes	+ 1
No	0
Was the method used to assess adverse effects described?	
Yes	+ 1
No	0
Was the method of statistical analysis described?	
Yes	+ 1
No	0

### Assessment of bias

The risk of bias in individual studies was divided into five parts: selection bias (random generation sequence and allocation concealment), performance and detection bias (blind), attrition bias (incomplete data), reporting bias (selective reporting), and other biases. Publication bias across studies would be shown by funnel plot if necessary.

### Statistical analysis

We analyzed continuous data by mean difference (MD) and its corresponding 95% confidence interval (CI). Odds ratio (OR) and its corresponding 95% CI were calculated for dichotomous data. We assessed heterogeneity by using the *I*^2^ statistic. *I*^2^ value above 50% was considered as high heterogeneity and a random-effects model would be used, while a value below 50% was considered as low heterogeneity and a fixed-effects model would be adopted [32]. Subgroup analyses would be considered when meeting high heterogeneity. Statistical analyses were performed using Review Manager 5.3 software. Forrest plots were used to describe the primary results of the meta-analysis. Funnel plots for primary outcomes (TBL and drain output) were generated to evaluate the potential publication bias. *P* value < 0.05 was considered statistically significant.

Formal ethical approval was deemed not necessary in our meta-analysis.

## Result

### Search results

**Fig. 1:**
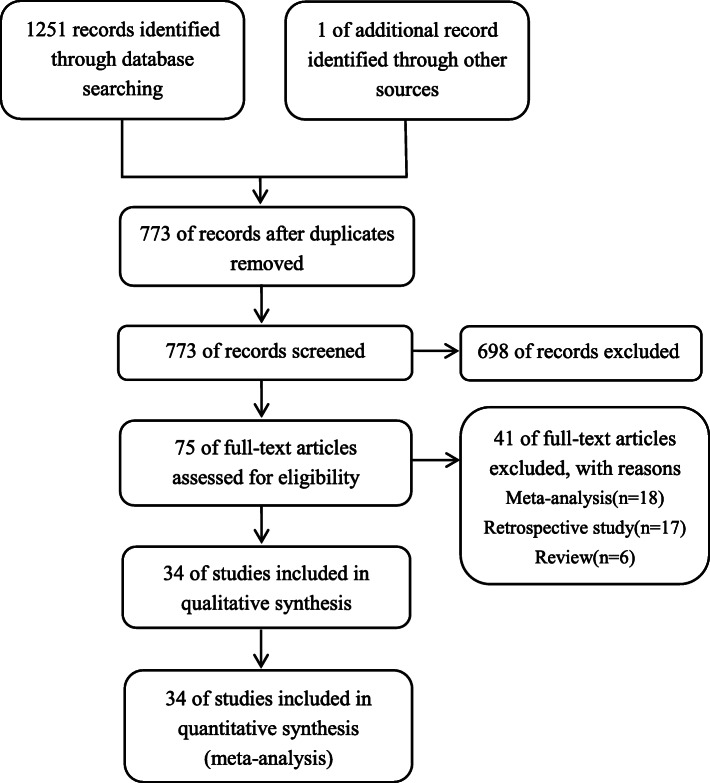
Flow diagram of the literature search

Figure 1 shows detailed steps of the literature search, in which 773 studies were reviewed: 698 studies were excluded after screening titles and abstracts, and the remaining 75 studies were reviewed in full text. After excluding 41 studies according to selection criteria, 34 studies encompassing 3867 patients were included in our study [13–26, 29, 33–51].

### Study characteristics and quality assessments

As shown in Table 2, the sample size of the included studies ranged from 25 to 320, and the mean age of patients ranged from 57 to 73. Nine of the studies (9/34, 26.5%) favored IA administration, while four of the studies (4/34, 11.8%) preferred IV administration.

**Table 2:** Characteristics of the study

Study	Design	Country	No. of patients		Age (years)		Sex (male/female)		BMI (kg/m^2^)		Follow-up	Conclusion
			IV	IA	IV	IA	IV	IA	IV	IA		
Jules-Elysee et al. [14]	RCT	USA	31	32	65.6 ± 8.4	65.0 ± 6.9	11/20	12/20	31.6 ± 7.1	31.1 ± 5.2	Unclear	IV > IA
Laoruengthana et al. [13]	RCT	Thailand	76	75	64.01 ± 7.68	64.81 ± 8.06	62/14	63/12	27.8 ± 5.2	27.6 ± 4.2	Unclear	IA > IV
Zhang et al. [15, 52]	RCT	China	50	50	63.12 ± 8.79	59.86 ± 12.01	12/38	10/40	23.9 ± 4.7	25.0 ± 4.3	6 months	IA > IV
Abdel et al. [20]	RCT	USA	320	320	66	67	127/193	133/187	31.3	31.6	Unclear	IV > IA
Ahmed et al. [23]	RCT	Pakistan	70	70	63.30 ± 9.51	64.39 ± 9.07	28/42	32/38	Unclear	Unclear	Unclear	IA > IV
López-Hualda et al. [21]	RCT	Spain	30	30	73.1 ± 7.3	72.9 ± 7.1	6/24	11/19	Unclear	Unclear	1 year	IA > IV
George et al. [16]	RCT	India	55	58	64.1	63.8	24/31	14/44	29.4	31.1	6 weeks	Neutral
Subramanyam et al. [19]	RCT	India	91	91	62.9 ± 6.8	62.7 ± 7.5	31/60	35/56	28.9	29.9	6 weeks	Neutral
Wei et al. [18]	RCT	China	32	32	66.47 ± 8.28	66.43 ± 7.69	14/18	16/16	32.4 ± 3.7	34.2 ± 5.0	3 months	Neutral
Goyal et al. [36]	RCT	Australia	85	83	68.8 ± 7.4	66.7 ± 8.9	40/47	38/43	30.3 ± 6.1	31.0 ± 5.3	1 month	Neutral
Lacko et al. [22]	RCT	Slovakia	30	30	68.4 ± 7.2	67.5 ± 7.7	12/18	13/17	31.1 ± 4.7	31.9 ± 4.7	3 months	IV > IA
Maniar et al. [33]	RCT	India	50	25	65.7 ± 7.6	62.2 ± 7.1	7/43	2/23	30.2 ± 4.5	30.3 ± 3.9	3 months	Neutral
Prakash et al. [26]	RCT	India	50	50	70.2	71	NR	NR	Unclear	Unclear	3 months	IV > IA
Song et al. [35]	RCT	South Korea	50	50	69.2 ± 6.4	69.8 ± 6.8	6/44	8/42	26.52 ± 3.3	26.96 ± 4.2	3 months	Neutral
Stowers et al. [24]	RCT	New Zealand	51	60	71 ± 8.6	70 ± 8.5	27/24	28/32	31.2 ± 5.5	31.2 ± 5.5	6 months	Neutral
Uğurlu et al. [34]	RCT	USA	40	42	69.4 ± 7.5	70.6 ± 8.6	11/29	9/33	30.8 ± 5.3	31.1 ± 5.4	10 days	Neutral
Wang et al. [11, 23]	RCT	China	50	50	67.42 ± 8.20	67.98 ± 5.97	14/36	14/36	26.7 ± 3.4	25.9 ± 3.8	5 weeks	IA > IV
Zekcer et al. [25]	RCT	Brazil	30	30	65.7	65.7	6/24	9/21	Unclear	Unclear	Unclear	Neutral
Aggarwal et al. [39]	RCT	India	35	35	58.77 ± 10.14	55.66 ± 8.71	13/22	12/23	26.33 ± 3.79	27.33 ± 4.63	6 months	IA > IV
Chen et al. [29, 53]	RCT	Singapore	50	50	65 ± 8	65 ± 8	15/35	10/40	28 ± 5	28 ± 7	1 month	Neutral
Drosos et al. [38]	RCT	Greece	30	30	69.27 ± 7.21	71.10 ± 6.32	6/24	6/24	32.79 ± 5.04	33.38 ± 6.08	1 month	Neutral
Keyhani et al. [42]	RCT	Iran	40	40	68.4 ± 10.4	67 ± 11.9	26/14	23/17	32.7 ± 5.5	31.3 ± 5.4	2 weeks	Neutral
May et al. [37]	RCT	USA	69	62	65.0 ± 9.6	63.0 ± 10.6	11/58	18/44	33.8	33.8	1 month	Neutral
Pinsornsak et al. [41]	RCT	Thailand	30	30	69.97 ± 7.55	67.63 ± 7.96	7/23	5/25	26.52 ± 3.7	27.96 ± 4.99	2 weeks	Neutral
Tzatzairis et al. [40]	RCT	Greece	40	40	69.55 ± 6.61	69.10 ± 8.68	9/31	7/33	32.60 ± 4.09	32.60 ± 4.50	6 weeks	Neutral
Aguilera et al. [43]	RCT	Spain	50	50	72.49 ± 7.68	72.53 ± 6.60	38/12	32/18	30.20 ± 4.10	30.89 ± 4.37	2 months	Neutral
Digas et al. [44]	RCT	Greece	30	30	70 ± 6.5	71 ± 7.0	2/28	7/23	Unclear	Unclear	1 year	IA > IV
Öztaş et al. [45]	RCT	Turkey	30	30	68.56	67.06	5/25	4/26	Unclear	Unclear	3 months	IA > IV
Gomez-Barrena et al. [46]	RCT	Spain	39	39	71.8 ± 10.3	70.1 ± 9.1	25/14	26/13	30.2 ± 4.2	30.4 ± 4.1	1 month	Neutral
Patel et al. [47]	RCT	USA	42	47	64.9 ± 7.8	64.8 ± 9.7	10/32	13/34	35.8 ± 8.6	32.7 ± 7.0	2 weeks	Neutral
Sarzaeem et al. [48]	RCT	Iran	50	100	66.9 ± 7.2	67.8 ± 7.2	7/43	13/87	31.6 ± 2.7	31.5 ± 3.4	Unclear	Neutral
Soni et al. [49]	RCT	India	40	40	69.05 ± 4.10	69.45 ± 4.71	19/21	17/23	Unclear	Unclear	6 weeks	Neutral
Seo et al. [50]	RCT	South Korea	50	50	66.8 ± 6.3	67.5 ± 6.6	6/44	5/45	28.1 ± 3.1	27.8 ± 3.5	2 months	IA > IV
Maniar et al. [51]	RCT	India	160	40	67.4 ± 8.1	67.4 ± 7.9	36/124	6/34	29.2 ± 5.4	30.9 ± 5.2	3 months	Neutral

*RCT* randomized controlled trial, *IV* intravenous group, *IA* intra-articular group, *TKA* total knee arthroplasty, *BMI* body mass index

Methods of administration and types of operation are presented in Table 3. One study (Maniar et al.) included four IV groups and another study (Maniar et al.) included two IV groups [33, 51]. One study (Sarzaeem et al.) had two IA groups with different dosages [48]. Unilateral TKA was performed in 31 studies (31/34, 91.2%) while bilateral TKA was performed in three studies (31/34, 8.8%). Twenty-four studies (24/34, 70.6%) adopted medial parapatellar, four (4/34, 11.8%) chose the midvastus approach, and two studies (2/34, 5.9%) used subvastus parapatellar, while the approach was unclear in the rest six studies (6/34, 17.6%).

**Table 3:** Methods of administration and operation

Study	IV dosage	IA dosage	Type of operation	Surgical approach
Jules-Elysee et al. [14]	1 g TXA × two doses; POPO	3 g TXA × one dose; before tourniquet release	Primary unilateral TKA	Unclear
Laoruengthana et al. [13]	10 mg/kg TXA × one dose; IO	15 mg/kg TXA × one dose; before closure	Primary unilateral TKA	Medial parapatellar
Zhang et al. [15, 52]	20 mg/kg TXA × one dose; PEO	3 g TXA × one dose; after closure	Primary unilateral TKA	Medial parapatellar
Abdel et al. [20]	1 g TXA × one dose; PEO	3 g TXA × one dose; after cemented	Primary unilateral TKA	Medial parapatellar or midvastus approach
Ahmed et al. [17]	1.5 g TXA × one dose; PTO	1.5 g TXA × one dose; while closure	Primary simultaneous bilateral TKA	Unclear
López-Hualda et al. [21]	1 g TXA × one dose; PEO	1 g TXA × one dose; after closure	Primary unilateral TKA	Medial parapatellar
George et al. [16]	10 mg/kg TXA × two doses; POPO	1.5 g TXA × one dose; before closure	Primary unilateral TKA	Medial parapatellar
Subramanyam et al. [19]	10 mg/kg TXA × one dose; PEO	1.5 g TXA × one dose; after closure	Primary unilateral TKA	Medial parapatellar
Wei et al. [18]	10 mg/kg TXA × one dose; PEO	1 g TXA × one dose; before tourniquet release	Primary unilateral TKA	Unclear
Goyal et al. [36]	1 g TXA × three doses; IO/PTO/PTO	3 g TXA × one dose; after closure	Primary unilateral TKA	Medial parapatellar
Lacko et al. [22]	10 mg/kg TXA × two doses; POPO	3 g TXA × one dose; after cemented	Primary unilateral TKA	Medial parapatellar
Maniar et al. [33]	10 mg/kg TXA × two doses (bilateral); IO	3 g TXA × two doses(bilateral); after cemented	Primary simultaneous bilateral TKA	Midvastus approach
	10 mg/kg TXA × three doses; POIOPO			
Prakash et al. [26]	10 mg/kg TXA × three doses; POIOPO	3 g TXA × one dose; before closure	Primary unilateral TKA	Medial parapatellar
Song et al. [35]	10 mg/kg TXA × three doses; POIOPO	1.5 g TXA × one dose; after closure	Primary bilateral TKA	Medial parapatellar
Stowers et al. [24]	1 g TXA × one dose; IO	1 g TXA × one dose; after closure	Primary unilateral TKA	Medial parapatellar
Uğurlu et al. [34]	20 mg/kg TXA × one dose; PEO	3 g TXA × one dose; after closure	Primary unilateral TKA	Medial parapatellar
Wang et al. [11, 23]	1 g TXA × one dose; IO	1 g TXA × one dose; before closure	Primary unilateral TKA	Medial parapatellar
Zekcer et al. [25]	20 mg/kg TXA × one dose; unclear	1.5 g TXA × one dose; before tourniquet release	Primary unilateral TKA	Unclear
Aggarwal et al. [39]	15 mg/kg TXA × two dose; IOPO	15 mg/kg TXA × one dose; before closure	Primary simultaneous bilateral TKA	Medial parapatellar
Chen et al. [29, 53]	1.5 g TXA × one dose; IO	1.5 g TXA × one dose; after cemented	Primary unilateral TKA	Medial parapatellar
Drosos et al. [38]	1 g TXA × one dose; PEO	1 g TXA × one dose; before closure	Primary unilateral TKA	Medial parapatellar
Keyhani et al. [42]	0.5 g TXA × one dose; IO	1.5 g TXA × two doses; before/after closure	Primary unilateral TKA	Medial parapatellar
May et al. [37]	1 g TXA × two doses; POPO	2 g TXA × one dose; after closure	Primary unilateral TKA	Unclear
Pinsornsak et al. [37]	0.75 mg TXA × one dose; IO	0.75 mg × one dose; before tourniquet release	Primary unilateral TKA	Medial parapatellar
Tzatzairis et al. [40]	1 g TXA × one dose; PEO	1 g TXA × one dose; after closure	Primary unilateral TKA	Medial parapatellar
Aguilera et al. [43]	1 g TXA × two doses; POIO	1 g TXA × one dose; after cemented	Primary unilateral TKA	Medial parapatellar
Digas et al. [44]	15 mg/kg TXA × one dose; IO	2 g TXA × one dose; after closure	Primary unilateral TKA	Medial parapatellar
Öztaş et al. [45]	15 mg/kg TXA × two doses; POPO 10 mg/kg TXA × one dose; 1-h infusion	2 g TXA × one dose; before tourniquet release	Primary unilateral TKA	Unclear
Gomez-Barrena et al. [46]	15 mg/kg TXA × two doses; IOPO	3 g TXA × one dose; before + after closure	Primary unilateral TKA	Medial parapatellar
Patel et al. [47]	10 mg/kg TXA × one dose; IO	2 g TXA × one dose; before tourniquet release	Primary unilateral TKA	Medial or subvastus parapatellar
Sarzaeem et al. [48]	1.5 g TXA × one dose; PTO	1.5 g TXA × one dose; after closure	Primary unilateral TKA	Subvastus approach
		3 g TXA × one dose; before closure		
Soni et al. [49]	10 mg/kg TXA × three doses; POIOPO	3 g TXA × one dose; before tourniquet release	Primary unilateral TKA	Midvastus approach
Seo et al. [50]	1.5 g TXA × one dose; PTO	1.5 g TXA × one dose; while closure	Primary unilateral TKA	Medial parapatellar
Maniar et al. [51]	10 mg/kg TXA × one dose; IO	3 g TXA × one dose; before tourniquet release	Primary unilateral TKA	Midvastus approach
	10 mg/kg TXA × two doses; IOPO			
	10 mg/kg TXA × two doses; POIO			
	10 mg/kg TXA × three doses; POIOPO			

*IO* intraoperative dose, *IOPO* intra- and postoperative doses, *PEO* preoperative dose, *POIO* pre- and intraoperative doses, *POIOPO* all three doses, *POPO* pre- and postoperative doses, *PTO* postoperative dose, *TXA* tranexamic acid

Table 4 summarizes the detailed surgical protocols. Low-molecular-weight heparin (LMWH) was the preferred prophylactic choice for thrombosis (21/34, 61.8%), following by pumping exercise and compression stocking (7/34, 20.6%), and aspirin (6/34, 17.6%). Both Doppler ultrasound and clinical examination were the most commonly used screening method for DVT (16/34, 47.1%), and chest CT was used in five studies (5/34, 14.7%), while nine (9/34, 26.5%) remained unclear. Cemented prosthesis was adopted in 27 studies (27/34, 79.4%), tourniquet was used in 31 studies (31/34, 91.2%), and 19 of the studies (31/34, 55.9%) clamped the drain tube after the operation.

**Table 4:** Surgical protocols

Study	Thromboprophylaxis	DVT screening method	Prosthetic properties	Blood transfusion protocol	Tourniquet	Drainage
Jules-Elysee et al. [14]	Unclear	Unclear	Cemented	Unclear	Yes	Clamped for 4 h
Laoruengthana et al. [13]	LMWH/warfarin	Unclear	Cemented	Hb < 9.0 g/L	Yes	Clamped for 3 h
Zhang et al. [15, 52]	Rivaroxaban	Doppler ultrasound	Cemented	Unclear	Yes	Unclear
Abdel et al. [20]	Aspirin/warfarin	Unclear	Cemented	Hb < 7.0 g/dL Hb < 10.0 g/dL + symptoms	Yes	Unclear
Ahmed et al. [17]	Unclear	Unclear	Unclear	Unclear	Unclear	Unclear
López-Hualda et al. [21]	Unclear	Unclear	Cemented	Hb < 8.0 g/dL + symptoms	Yes	Unclear
George et al. [16]	LMWH/aspirin	Doppler ultrasound	Cemented	Hb < 7.0 g/dL	Yes	Unclear
Subramanyam et al. [19]	Aspirin Calf pump	Clinical examination Doppler ultrasound	Cemented	Hb < 8.0 g/dL Hb < 10.0 g/dL + symptoms	Yes	No drain
Wei et al. [18]	LMWH	Unclear	Cemented	Hb < 8.0 g/dL Hb < 10.0 g/dL + symptoms	Yes	Unclear
Goyal et al. [36]	LMWH/aspirin Compression stocking	Doppler ultrasound	Hybrid	Hb < 7.0 g/dL Hb < 10.0 g/dL + symptoms	No	Closed
Lacko et al. [22]	Unclear	Doppler ultrasound	Cemented	Hb < 8.0 g/dL Hb < 9.0 + symptoms	Yes	Unclear
Maniar et al. [33]	LMWH Ankle pumping exercise Compression stocking	Clinical examination Doppler ultrasound	Cemented	Hn < 8.5 g/dL Hb < 10.0 g/dL + symptoms	Yes	Clamped for 2 h
Prakash et al. [26]	LMWH Calf pump	Doppler ultrasound Chest CT	Cemented	Hb < 8.0 g/dL	Yes	Clamped for 30 min
Song et al. [35]	LMWH in high-risk patient	Doppler ultrasound Chest CT	Cemented	Hb < 8.0 g/dL	Yes	Clamped for 10 min
Stowers et al. [24]	Aspirin	Clinical examination	Cemented	Hb < 8.0 g/dL Hb < 10.0 g/dL + symptoms	Yes	No drain
Uğurlu et al. [34]	LMWH Compression stocking	Clinical examination	Unclear	Hb < 8.0 g/dL Hb > 8.0 g/dL + symptoms	Yes	Clamped for 1 h
Wang et al. [11, 23]	LMWH/rivaroxaban Elastic bandage	Doppler ultrasound	Cemented	Hb < 6.0 g/dL Hb > 6.0 + symptoms	Yes	Clamped for 2 h
Zekcer et al. [25]	LMWH Compression stocking	Unclear	Cemented	Hb < 8.0 g/dL	Yes	Unclear
Aggarwal et al. [39]	Aspirin	Clinical examination	Cemented	Hb > 8.0 g/dL + symptoms	Yes	Clamped for 1 h
Chen et al. [29, 53]	LMWH Calf pumps	Clinical examination Doppler ultrasound Chest CT	Cemented	Hb < 8.0 g/dL Hb < 10.0 g/dL + symptoms	Yes	Unclear
Drosos et al. [38]	LMWH Compression stocking	Clinical examination Doppler ultrasound	Hybrid	Hb < 10.0 g/dL + symptoms	Yes	No clamp
Keyhani et al. [42]	LMWH	Doppler ultrasound	Cemented	Hb < 8.0 g/dL	Yes	Clamped for 2 h
May et al. [37]	LMWH Sequential compression	Clinical examination	Unclear	Hb < 7.0 g/dL Hb < 10.0 g/dL + symptoms	Yes	No drain
Pinsornsak et al. [37]	Ankle pumping exercise Early ambulation	Clinical examination	Cemented	Hb < 10.0 g/dL + symptoms	Yes	Clamped for 3 h
Tzatzairis et al. [40]	LMWH Compression stocking	Doppler ultrasound Clinical examination Chest CT	Cemented	Hb < 10.0 g/dL + symptoms	No	Clamped for 1 h
Aguilera et al. [43]	LMWH	Clinical examination	Cemented	Hb < 8.0 g/dL Hb < 9.0 g/dL + symptoms	Yes	Clamped for 1 h
Digas et al. [44]	Tinzaparin	Clinical examination	Cemented	Hb < 8.5 g/dL Hb < 9.5 g/dL + symptoms	Yes	Clamped for 3 h
Öztaş et al. [45]	LMWH	Clinical examination	Unclear	Hb < 8.0 g/dL Hb < 10.0 g/dL + symptoms	Yes	Clamped for 30 min
Gomez-Barrena et al. [46]	LMWH	Clinical examination Doppler ultrasound	Cemented	Hb < 8.0 g/dL Hb < 10.0 g/dL + symptoms	Yes	Clamped for 2 h
Patel et al. [47]	LMWH	Doppler ultrasound Chest CT	Unclear	Hb < 8.0 g/dL + symptoms	Yes	Yes
Sarzaeem et al. [48]	Unclear	Unclear	Cemented	Hb < 8.0 g/dL Hb < 10.0 g/dL + symptoms	Yes	Clamped for 1 h
Soni et al. [49]	LMWH Ankle pumping exercise	Clinical examination	Cemented	Hb < 8.0 g/dL	Yes	Clamped for 1 h
Seo et al. [50]	Unclear	Unclear	Cemented	Hb < 8.0 g/dL Hb < 10.0 g/dL + symptoms	Yes	No drain
Maniar et al. [51]	LMWH Ankle pumping exercise Compression stocking	Clinical examination Doppler ultrasound	Cemented	Hb < 8.5 g/dL Hb < 10.0 g/dL + symptoms	Yes	Clamped for 2 h

*Hb* hemoglobin, *LMWH* low-molecular-weight heparin

Quality assessment and assessment of bias are presented in Table 5. In all, 25 studies (25/34, 73.5%) are high-quality and nine (9/34, 26.5%) are moderate-quality evidences.

**Table 5:** Methodological quality of included studies

Study	Quality score	Random generation sequence	Allocation concealment	Blind	Incomplete outcome data	Selective reporting	Other biases
Jules-Elysee et al. [14]	7	Computer-generated randomization schedule	Unclear	Yes	No	No	No
Laoruengthana et al. [13]	8	Computer-generated numbers	Concealed envelope	Yes	No	No	No
Zhang et al. [15, 52]	8	Randomized numbers table	Labeled with numbering code	Yes	No	No	No
Abdel et al. [20]	6	Randomized but unknown method	Unclear	Yes	No	No	No
Ahmed et al. [17]	6	The lottery method	Unclear	No	No	No	No
López-Hualda et al [21]	5	Randomized but unknown method	Unclear	No	No	No	No
George et al. [16]	8	Computer-generated numbers	Concealed envelope	Yes	No	No	No
Subramanyam et al. [19]	8	Computer-generated numbers	Concealed envelope	Yes	No	No	No
Wei et al. [18]	8	Randomized numbers table	Concealed envelope	Yes	No	No	No
Goyal et al. [36]	8	Computer-generated numbers	Concealed envelope	Yes	No	No	No
Lacko et al. [22]	6	Computer-generated numbers	Unclear	No	No	No	No
Maniar et al. [33]	8	Randomly drawing sealed envelope from container	Concealed envelope	Yes	No	No	No
Prakash et al. [26]	7	Randomized but unknown method	Concealed envelope	Yes	No	No	No
Song et al. [35]	8	Computer-generated numbers	Concealed envelope	Yes	No	No	No
Stowers et al. [24]	8	Block randomization	Concealed envelope	Yes	No	No	No
Uğurlu et al. [34]	5	Randomized but unknown method	Unclear	No	No	No	No
Wang et al. [11, 23]	8	Randomly drawing sealed envelope from container	Concealed envelope	Yes	No	No	No
Zekcer et al. [25]	8	Randomly drawing sealed envelope from container	Concealed envelope	Yes	No	No	No
Aggarwal et al. [39]	8	Computer-generated numbers	Concealed envelope	Yes	No	No	No
Chen et al. [29, 53]	8	Randomized numbers table	Concealed envelope	Yes	No	No	No
Drosos et al. [38]	8	Stratified randomization by minimization	Concealed envelope	Yes	No	No	No
Keyhani et al. [42]	5	Randomized but unknown method	Unclear	No	No	No	No
May et al. [37]	7	Randomized numbers table	Unclear	Yes	No	No	No
Pinsornsak et al. [37]	7	Randomized but unknown method	Concealed envelope	Yes	No	No	No
Tzatzairis et al. [40]	6	Stratified randomization by minimization	Unclear	No	No	No	No
Aguilera et al. [43]	7	Randomized numbers table	Unclear	Yes	No	No	No
Digas et al. [44]	7	Randomized but unknown method	Concealed envelope	Yes	No	No	No
Öztaş et al. [45]	5	Randomized but unknown method	Unclear	No	No	No	No
Gomez-Barrena et al. [46]	7	Randomized but unknown method	Concealed envelope	Yes	No	No	No
Patel et al. [47]	7	Excel’s randomization	Unclear	Yes	No	No	No
Sarzaeem et al. [48]	7	Randomized numbers table	Unclear	Yes	No	No	No
Soni et al. [49]	6	Computer-generated numbers	Unclear	No	No	No	No
Seo et al. [50]	7	Randomized numbers table	Unclear	Yes	No	No	No
Maniar et al. [51]	8	Randomly drawing sealed envelope from container	Concealed envelope	Yes	No	No	No

### Meta-analysis of outcomes

All the results are listed in Table 6, including primary outcomes, secondary outcomes, three subgroup analyses, and three low heterogeneity analyses.

**Table 6:** Results of meta-analysis and subgroup analyses

Variables	Studies (*n*)	Patients (*n*)	*P* value	Incidence: OR/MDs (95% CI)	Heterogeneity: *P* value (*I*^2^)	Model
Total blood loss (TBL)	18	1656	< 0.001*	63.99 (27.81 to 100.16)	< 0.001* (81%)	Random
	13	1197	< 0.001*	33.38 (19.24 to 47.51)	0.34 (11%)	Fixed
Drain output	17	1494	0.03*	28.44 (2.61 to 54.27)	< 0.001* (93%)	Random
Clamp < 2 h	7	607	0.03*	51.47 (6.02 to 96.92)	< 0.001* (92%)	Random
Clamp ≥ 2 h	10	887	0.51	12.40 (− 24.85 to 49.65)	< 0.001* (89%)	Random
Hidden blood loss (HBL)	6	640	0.83	7.57 (− 60.34 to 75.47)	0.006 (69%)	Random
Hemoglobin (Hb) fall	19	1749	0.79	− 0.02 (− 0.20 to 0.16)	< 0.001* (87%)	Random
POD1	10	1052	0.07	− 0.34 (− 0.70 to 0.02)	< 0.001* (91%)	Random
	8	839	0.86	− 0.01 (− 0.11 to 0.13)	0.14 (36%)	Fixed
POD2	8	701	0.37	0.17 (− 0.20 to 0.53)	<0.001* (82%)	Random
	6	531	0.36	− 0.08 (− 0.25 to 0.09)	0.11 (44%)	Fixed
POD3+	6	637	0.001*	0.24 (0.09 to 0.39)	0.18 (34%)	Fixed
Transfusion rate	25	2950	0.62	0.93 (0.69 to 1.24)	0.54 (0%)	Fixed
Complications	20	2594	0.98	1.00 (0.72 to 1.39)	0.47 (0%)	Fixed
DVT	10	1641	0.83	0.92 (0.44 to 1.92)	0.84 (0%)	Fixed
PE	3	342	0.98	1.02 (0.25 to 4.20)	0.81 (0%)	Fixed
Wound complications	14	1465	0.83	0.95 (0.58 to 1.55)	0.39 (6%)	Fixed
Other adverse events	13	1899	0.69	1.10 (0.68 to 1.80)	0.42 (2%)	Fixed
Length of stay	7	748	0.33	0.07 (− 0.07 to 0.22)	0.35 (11%)	Fixed
Tourniquet time	9	816	0.19	− 1.22 (− 3.06 to 0.62)	0.74 (0%)	Fixed

*POD* postoperative day, *DVT* deep vein thrombosis

*≤ 0.05

#### Total blood loss

**Fig. 2:**
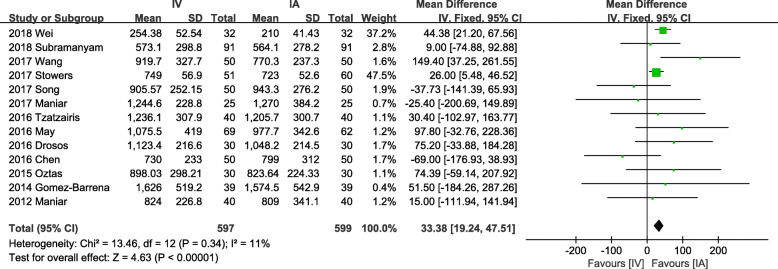
Forest plot showing low heterogeneity effect of IV vs IA TXA on total blood loss

**Fig. 3:**
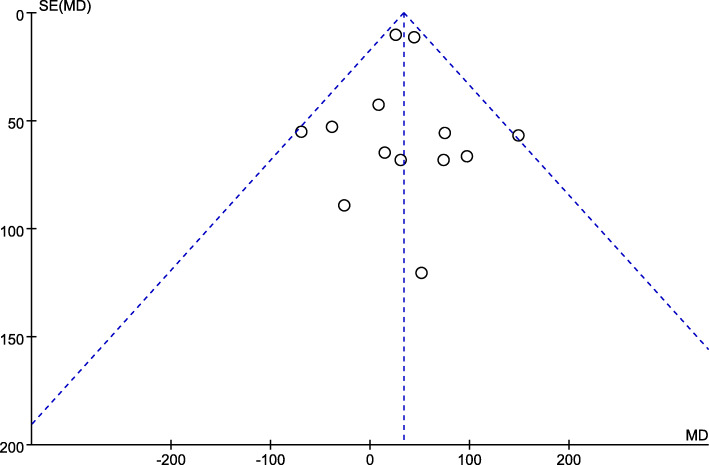
Funnel plot of TBL shows low publication bias

Eighteen studies provided valid data of TBL on 1656 patients. Given the presence of significant heterogeneity among studies (*P* < 0.001, *I*^2^ = 81%), we used a random-effects model for analysis. IA administration showed a significant advantage compared to IV administration (MD = 63.99, 95% CI = 27.81 to 100.16, *P* < 0.001). Concerning about the high heterogeneity, we performed a sensitivity analysis based on the risk of bias and got another lower heterogeneity result (Fig. 2) by analyzing 13 studies (*P* = 0.34, *I*^2^ = 11%) with a fixed-effects model, which still revealed a significant superiority of IA administration (MD = 33.38, 95% CI = 19.24 to 47.51, *P* < 0.001). Publication bias is shown by a funnel plot (Fig. 3).

#### Drain output

**Fig. 4:**
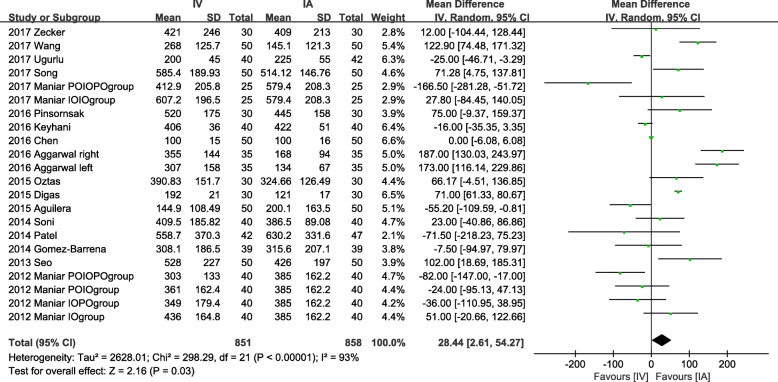
Forest plot showing the effect of IV vs IA TXA on drain output

Seventeen studies involving 1494 patients provided valid data of drain output. Due to significant heterogeneity among studies (*P* < 0.001, *I*^2^ = 93%), we used a random-effects model for analysis. IA administration showed a significant advantage (Fig. 4) compared to IV administration (MD = 28.44, 95% CI = 2.61 to 54.27, *P* = 0.03).

Drainage volume was analyzed in subgroup based on the duration of tube clamping. For studies in which the drainage tube was clamped postoperatively less than two hours, a significant superiority was shown in the IA group compared to the IV group (MD = 51.47, 95% CI = 6.02 to 96.92, *P* = 0.03). Considering the high heterogeneity (*P* < 0.001, *I*^2^ = 92%), a random-effects was used for analysis. There was no significant difference (MD = 12.40, 95% CI = − 24.85 to 49.65, *P* = 0.51) for studies in which the drainage tube was clamped postoperatively over 2 h with high heterogeneity (*P* < 0.001, *I*^2^ = 89%).

#### Hidden blood loss

Only six studies including 640 patients reported HBL. Since there existed significant heterogeneity among studies (*P* = 0.006, *I*^2^ = 69%), we used a random-effects model for analysis. There existed no significant difference between the IV and IA groups (MD = 7.57, 95% CI = − 60.34 to 75.47, *P* = 0.83) on HBL.

#### Hemoglobin fall

In all, 19 studies involving 1749 patients reported the data of postoperative Hb fall. Because different studies reported Hb of postoperative day (POD) 1 to 5 with high heterogeneity (*P* < 0.001, *I*^2^ = 87%), we conducted subgroup analyses based on POD1, POD2, or POD3+.

Ten studies involving 1052 patients reported the POD1 Hb fall. The random-effects model (*P* < 0.001, *I*^2^ = 91%) was used for analysis, and there was no significant difference between the IV and IA groups (MD = − 0.34, 95% CI = − 0.70 to 0.02, *P* = 0.07). Regarding the high heterogeneity, a sensitivity analysis was performed and two studies were excluded [14, 48], then we got a lower heterogeneity result (*P* = 0.14, *I*^2^ = 36%) by analyzing the rest of 8 studies including 839 patients with a fixed-effects model. No significant difference was shown between the IV and IA groups (MD = − 0.01, 95% CI = − 0.11 to 0.13, *P* = 0.86).

Eight studies involving 701 patients reported the POD2 Hb fall. Considering the significant heterogeneity among studies (*P* < 0.001, *I*^2^ = 82%), we used a random-effects model for analysis. There existed no significant difference between the IV and IA groups (MD = 0.17, 95% CI = − 0.20 to 0.53, *P* = 0.37). We also performed a sensitivity analysis based on the risk of bias and excluded two studies [23, 39] and got a lower heterogeneity (*P* = 0.11, *I*^2^ = 44%) result by analyzing the rest of six studies involving 531 patients with a fixed-effects model. No significant difference was shown between the IV and IA groups (MD = − 0.08, 95% CI = − 0.25 to 0.09, *P* = 0.36).

Six studies involving 637 patients reported the POD3+ Hb fall. Because of low heterogeneity among studies (*P* = 0.18, *I*^2^ = 34%), a fixed-effects model was used for analysis. The IA group showed a significant advantage compared to the IV group (MD = 0.24, 95% CI = 0.09 to 0.39, *P* = 0.001).

#### Blood transfusion rate

Twenty-eight studies involving 3270 patients had data on blood transfusion. Transfusions were reported as 109/1664 (6.6%) in the IV group and 99/1606 (6.2%) in the IA group. Only 25 studies with 2950 patients were included in our meta-analysis, while the other three studies reported no transfusion event. The risk of a blood transfusion was similar between the two groups (OR = 0.93, 95% CI = 0.69 to 1.24, *P* = 0.62), and the data showed low heterogeneity (*P* = 0.54, *I*^2^ = 0%).

#### Complications

In our study, certain complications were our concern, including DVT, PE, wound complications, and other adverse events. In all, 33 studies involving 3807 patients mentioned data of complications. The incidence of complications was mentioned as 77/1946 (4.0%) in the IV group and 77/1861 (4.1%) in the IA group. In these 33 studies, 13 of them reported no complication, so only 20 studies with 2594 patients were included in the meta-analysis. The risk was the same between the two groups (OR = 1.00, 95% CI = 0.72 to 1.39, *P* = 0.98) with low heterogeneity (*P* = 0.47, *I*^2^ = 0%).

In subgroup analysis, complications were classified into four types: DVT, PE, wound complications, and other adverse events. All subgroups showed insignificant differences between the IV and IA groups.

There were 23 DVT events reported in ten studies among all 33 studies. Pooled results showed a similar risk (OR = 0.92, 95% CI = 0.44 to 1.92, *P* = 0.83) with low heterogeneity (*P* = 0.84, *I*^2^ = 0%). Both the IV and IA groups had four PE events reported in three studies [15, 24, 37]. The risk of PE was similar between the IV group and IA group (OR = 1.02, 95% CI = 0.25 to 4.20, *P* = 0.98) with low heterogeneity (*P* = 0.81, *I*^2^ = 0%).

Wound complications included infection, necrosis, delay healing, and dehiscence. There were 58 wound complications reported in 14 studies. A fixed-effects model was used due to low heterogeneity (*P* = 0.39, *I*^2^ = 6%), and a similar risk of wound complications was shown in two groups (OR = 0.95, 95% CI = 0.58 to 1.55, *P* = 0.83 ).

Other adverse events were reported in 65 patients of 13 studies. Zhang et al. [15] reported 14 patients with idiopathic venous thromboembolism, and Wang et al. [23] reported one patient with intramuscular vein thrombosis. Besides, Abdel et al. [20] reported one patient with a thrombotic cerebrovascular accident. Functional disorders, such as stiffness, vomiting, nausea, dizziness, constipation, and paresthesia, were also reported in several studies [36, 43, 46]. A similar risk was shown (OR = 1.10, 95% CI = 0.68 to 1.80, *P* = 0.69) with low heterogeneity (*P* = 0.42, *I*^2^ = 2%) between IA and IV.

#### Length of hospital stay

Seven studies involving 748 patients reported data on length of hospital stay. Because of low heterogeneity (*P* = 0.35, *I*^2^ = 11%), we used a fixed-effects model for analysis. There was no significant difference in this comparison (MD = 0.07, 95% CI = − 0.07 to 0.22, *P* = 0.33).

#### Duration of tourniquet application

Nine studies including 815 patients reported data of tourniquet time. A fixed-effects model was used for analysis due to the low heterogeneity (*P* = 0.74, *I*^2^ = 0%). It did not show a statistical difference between the two groups (MD = − 1.22, 95% CI = − 3.06 to 0.62, *P* = 0.19).

## Discussion

The most important finding in our study is that the difference of TBL and drain output between IV and IA administration is supported by newly added RCTs. Based on available evidences, the IA group shows significant superiority over the IV group regarding TBL, drain output, and POD3+ Hb fall. Besides, this study suggests that there exists no statistical difference on HBL, POD1 and POD2 Hb fall, incidence of blood transfusion, length of hospital stay, and time of tourniquet application between the two groups.

As an antifibrinolytic agent, TXA is a synthetic derivative of the amino acid lysine which competitively blocks the lysine-binding sites in the plasmin and plasminogen activator molecules, thereby preventing dissolution of the fibrin clot [54]. A previous study [6], which included 23,236 patients undergoing primary TKA, proved that TXA application was associated with decreased blood loss and transfusion risk without noticeably increased risk of complications. Besides, it could also reduce the risk of venous thromboembolism [6]. Several previous studies have compared IV and IA administration in TKA: Xie et al. [55] included 18 RCTs and found no significant difference between IV and IA. Gianakos et al. [12] included 18 RCTs and 5 non-RCTs, and they found significant differences regarding TBL and drain output between IV and IA. However, it was a study of high heterogeneity. Therefore, we performed this meta-analysis with more newly published RCTs. Moreover, subgroup analysis and sensitivity analysis were performed to reach a more convincing conclusion.

In our study, IA administration shows significant superiority on the TBL to IV group (MD = 33.38, *P* < 0.001). A previous study indicated easier administration of topical TXA with a maximum concentration at the bleeding site and minimal systemic absorption [53], and therefore, topical application may deliver better blood loss control theoretically. The IA group also shows significant superiority on drain output (MD = 28.44, *P* = 0.03). The difference is more significant when the drainage tube is clamped postoperatively less than 2 h (MD = 51.47, *P* = 0.03). However, when the drainage tube is clamped over 2 h after surgery, there exists no statistical difference between them (*P* = 0.51). It is possibly due to a higher concentration of TXA and longer contact time in the IA approach.

There exists a significant difference on POD3+ Hb fall (MD = 0.24, *P* = 0.001), while POD1 (*P* = 0.86) and POD2 Hb fall (*P* = 0.36) show no noticeable difference between the two groups. POD3+ Hb fall is usually caused by HBL [55]. However, due to the limited data, there exists no difference on HBL (*P* = 0.83). Besides, IV administration of TXA has a maximal systemic absorption which may result in a shorter efficacy time in theory [56]. Therefore, it is a reasonable explanation of similar effects on POD1 and POD2, and a better result in the IA group on POD3+.

Fillingham et al. [5] published a clinical guideline of TXA application in joint replacement, but no optimal approach was recommended. In contrast, in our study, IA was found to be of superior value in light of the recently published RCT results. Although we have not compared IA with oral or combined administration, future clinical trials might validate our findings and possibly influence the revision of the clinical guideline of TXA. Besides, Fillingham et al. [5] also admitted dosage amount and multiple doses of TXA did not significantly affect the blood loss. However, several recent studies had different conclusions. Tzatzairis et al. [57] made a comparison between one to three doses of 15 mg/kg TXA intravenously and concluded that the three-dose group displayed better outcome. Lei et al. [58] reach the same conclusion by comparing 20 mg/kg and 60 mg/kg TXA intravenously. Moreover, Zhang et al. [52] even reported a better outcome of six-dose IV TXA. Besides, Tammachote et al. [59] compared high dosage (3 g) with low dosage (0.5 g) for IA TXA and also found a better outcome of high dosage. All the results of recent RCTs favor high-dose administration of TXA. Although TXA dosage and timing were popular topics, there is no meta-analysis about them by now. In our meta-analysis, there existed no standard dosage protocol for included studies (Table 3): In the IV group, 52.9% of the studies (18 studies) used a weight-based dosage (10 to 20 mg/kg) and the rest 47.1% of the studies (16 studies) chose a standard dosage (0.5 to 1.5 g). In the IA group, only 5.9% of the studies (2 studies) used a weight-based dosage (15 mg/kg) and the rest 94.1% of the studies (32 studies) chose a standard dosage (0.75 to 3 g). However, restricted by limited data, we did not perform a subgroup analysis for TXA dose and timing.

Advantages of our study include substantial high-quality RCTs (Table 5) and adequate analysis. 73.5% of the studies (25 studies) have detailed random generation sequence, and 55.9% of the studies (19 studies) have adequate allocation concealment. Besides, 73.5% of the studies (25 studies) are recent studies (published after 2015). Our analyzing methods are subgroup analysis and sensitivity analysis when the previous analysis has high heterogeneity.

There are several limitations in our studies. Firstly, the inherent bias in different studies because of the inconsistent threshold for blood transfusion cannot be overlooked. Besides, the DVT rate might be influenced by the inclusion criteria, and the RCT of TXA in a DVT high-risk population might be required to validate our findings. Furthermore, repeated dose seemed a better choice than a single dose in both IV and IA administration [52, 57–59], and therefore, different methods of administration may influence the result. Lastly, data for HBL, length of hospital stay, and duration of tourniquet application are limited for analysis, and cost-effectiveness remains to be investigated.

## Conclusion

IA administration of TXA is superior to IV TXA in patients receiving primary TKA regarding the performance on TBL, drain output, and POD3+ Hb fall, without noticeably increased risk of complications. Therefore, IA administration should be the preferred approach in clinical practice.

## Data Availability

We state that the data will not be shared because all the raw data are present in the figures included in the article.

## Abbreviations

IA: Intra-articular
IV: Intravenous
TXA: Tranexamic acid
TKA: Total knee arthroplasty
Hb: Hemoglobin
OA: Osteoarthritis
TBL: Total blood loss
HBL: Hidden blood loss
POD: Postoperative day
DVT: Deep vein thrombosis
PE: Pulmonary embolism
LMWH: Low-molecular-weight heparin
RCT: Randomized controlled trial
BMI: Body mass index
OR: Odds ratio
MD: Mean difference
CI: Confidence interval
